# The Rocky Road From Fed-Batch to Continuous Processing With *E. coli*

**DOI:** 10.3389/fbioe.2019.00328

**Published:** 2019-11-20

**Authors:** Julian Kopp, Christoph Slouka, Oliver Spadiut, Christoph Herwig

**Affiliations:** ^1^Christian Doppler Laboratory for Mechanistic and Physiological Methods for Improved Bioprocesses, Vienna, Austria; ^2^Research Area Biochemical Engineering, Institute of Chemical Engineering, Vienna, Austria

**Keywords:** *E. coli*, continuous processing, process understanding, burden reduction, from batch to continuous manufacturing

## Abstract

*Escherichia coli* still serves as a beloved workhorse for the production of many biopharmaceuticals as it fulfills essential criteria, such as having fast doubling times, exhibiting a low risk of contamination, and being easy to upscale. Most industrial processes in *E. coli* are carried out in fed-batch mode. However, recent trends show that the biotech industry is moving toward time-independent processing, trying to improve the space–time yield, and especially targeting constant quality attributes. In the 1950s, the term “chemostat” was introduced for the first time by Novick and Szilard, who followed up on the previous work performed by Monod. Chemostat processing resulted in a major hype 10 years after its official introduction. However, enthusiasm decreased as experiments suffered from genetic instabilities and physiology issues. Major improvements in strain engineering and the usage of tunable promotor systems facilitated chemostat processes. In addition, critical process parameters have been identified, and the effects they have on diverse quality attributes are understood in much more depth, thereby easing process control. By pooling the knowledge gained throughout the recent years, new applications, such as parallelization, cascade processing, and population controls, are applied nowadays. However, to control the highly heterogeneous cultivation broth to achieve stable productivity throughout long-term cultivations is still tricky. Within this review, we discuss the current state of *E. coli* fed-batch process understanding and its tech transfer potential within continuous processing. Furthermore, the achievements in the continuous upstream applications of *E. coli* and the continuous downstream processing of intracellular proteins will be discussed.

## Introduction

Using equipment at maximum capacity in long-term, quasi-perpetual processes is the dream of any industrial application in biotechnology (Plumb, 2005; Burcham et al., 2018). Many parts of the chemical industry (especially the petrochemical one) are already producing at a continuous level (Glaser, 2015). Continuous production in white biotechnology is also already implemented (Scholten et al., 2009; Kralisch et al., 2010; Luttmann et al., 2015); it is no huge surprise, therefore, that the red biopharmaceutical industry is trying to move toward “continuous processing” (Plumb, 2005; Gutmann et al., 2015; Burcham et al., 2018). Even though biopharmaceutical production is generally still carried out batch-wise, new technology has emerged (Walsh, 2014, 2018), and many drugs can currently be sold at the cheapest prices ever (Berlec and Strukelj, 2013; Jungbauer, 2013; Walsh, 2018). Continuous systems are already partially implemented in red biotechnology. Until now, however, these processes have only been carried out with mammalian cells (Vogel et al., 2012; Karst et al., 2017; Steinebach et al., 2017; Burgstaller et al., 2018). By switching from a batch-wise to a continuous production system, cultivations can be improved, which affects the duration of the run and thereby reduces the frequency of required setup and cleaning times. Furthermore, by switching to continuous processing, small footprint facilities can be implemented, leading to an increase in the overall space–time yield (Seifert et al., 2012; Bieringer et al., 2013; Lee et al., 2015; Adamo et al., 2016).

Looking at the technical realization of a continuous process, the upstream is regarded as rather simple. Chemostat processing, developed back in the 1950s (Novick and Szilard, 1950a), is well-known. Stable process parameters can be adjusted, and the bleed containing the desired amount of product can be subtracted from the culture vessels and further processed. However, the implementation of the downstream is highly dependent on the location of the product (Jungbauer, 2013; Slouka et al., 2018b). Fusing purification steps into an overall non-stop process seemed unlikely to be achieved a couple of years ago (Rathore, 2015; Vemula et al., 2015; Kante et al., 2018, 2019; VKR et al., 2019). Continuous chromatography in particular created a bottleneck; however, simulated moving bed chromatography (SMBC) improved significantly in terms of performance (Ötes et al., 2017, 2018). With state of the art technology, we are able to process proteins independent of location and at a continuous level (Jungbauer, 2013; Saraswat et al., 2013; Wellhoefer et al., 2013, 2014), and we are able to unite all process unit operations into one overall process (Lee et al., 2015; Zydney, 2015; Burcham et al., 2018).

With emerging process technology, the regulatory authorities evolved as well, and Food and Drug Administration (= FDA) regulations for the release of a product are clearly set (Gassman et al., 2017). Batch-wise production made it rather easy to break down product streams into certain product pools as upstream and downstream applications could be clearly separated and approved by the quality control (Lee et al., 2015). To enable continuous processing from a regulatory point of view, the process has to be separated into different lot numbers, creating a batch-wise system in itself (Jungbauer, 2013) to fulfill acceptance criteria by regulatory authorities (Gassman et al., 2017). As an integrated continuous process can achieve time-independent constant critical quality attributes (= CQAs) (Herwig et al., 2015; Zydney, 2015), the realization of such a process might even shorten the time-to-market. The first product derived from continuous production after its approval was produced in 2015, namely Orkambi (Matsunami et al., 2018). Prezista, being produced at a continuous level by Jansen, was accepted by the FDA in 2016 (Nasr et al., 2017). Ever since first approvals for continuously produced products emerged (Yang et al., 2017; Balogh et al., 2018), companies have tended to invest more into production lines so as to be capable of producing at a continuous level.

Still, the use of bacteria and yeast in continuous production modes is not common and has only been implemented once on an industrial scale for insulin production in *Saccharomyces cerevisiae* back in the 90s (Diers et al., 1991). State of the art recombinant protein production (RPP) in *E. coli* is still carried out in a fed-batch mode, and, with ongoing technology development, very high product titers can be achieved (Kopp et al., 2017; Wurm et al., 2017a; Hausjell et al., 2018; Slouka et al., 2018a). Still, “low-cost” biopharmaceuticals produced in bacteria are expensive to produce (Jia and Jeon, 2016); one gram of IPTG (Isopropyl-β-D-thiogalactopyranosid, currently purchased from Sigma-Aldrich for 76.8 €), is more expensive than one gram of 900 carat gold (currently worth 37.13 €). Therefore, a continuous production model for *E. coli* would be highly beneficial, increasing the overall space–time yield (Seifert et al., 2012; Bieringer et al., 2013). Counter-intuitively, the upstream section, which is technically easy to realize, thwarts industry plans as chemostat cultivations producing recombinant proteins within microbial hosts lack major instabilities (Diers et al., 1991); shifts in the transcriptome and proteome leads to enhanced acetate production, which seems to disturb RPP and decrease the overall specific productivity (q_p_) as a consequence (Peebo et al., 2015; Peebo and Neubauer, 2018). As *E. coli* still is a beloved workhorse for industry as well as for research (Rosano et al., 2019), we, within this review, highlight recent achievements in fed-batch processing and the first steps that have been implemented for the ongoing transition toward a continuous recombinant production system.

Additionally, the first achievements in the continuous upstream applications of *E. coli*; and the continuous downstream processing of intracellular proteins are discussed.

## The Current State of *E. coli* fed-Batch Process Understanding and its Tech Transfer Potential Within Continuous Processing

Process parameters and their effects on critical quality attributes are well-understood in *E. coli* fed-batch cultivations (Ferrer-Miralles et al., 2009; Babaeipour et al., 2015; Gupta and Shukla, 2017; Hausjell et al., 2018; Kante et al., 2018); they lead to high titers and predictable manufacturing (Slouka et al., 2018a,b). Still, RPP causes stress phenomena in the host cells, leading to decreased host capacity and, consequently, decreased growth rates (Rozkov et al., 2004; Silva et al., 2012; Ceroni et al., 2015; Kopp et al., 2017). Many recent publications revealed that RPP in *E. coli* suffers from a metabolic burden. Therefore, within this section we discuss the effects of metabolic burden on RPP and the determination thereof; intracellular vs. extracellular RPP; and enablers for continuous processing.

### Recombinant Protein Production in *E. coli* and Its Effect on Metabolic Burden

#### Metabolic Burden and Its Effects on Growth Rate

Recombinant protein production (RPP) has always been referred to as exhibiting a high metabolic burden onto the host cells (Heyland et al., 2011; Silva et al., 2012; Ceroni et al., 2015; Dvorak et al., 2015). Effects such as a decrease in the specific growth rate (= μ) and the specific substrate uptake rate (= q_s_) over cultivation time have been observed (Scott et al., 2010; Shachrai et al., 2010; Reichelt et al., 2017a,b). As higher amounts of intracellular proteins can be produced by applying higher feeding rates (Boström et al., 2005; Ukkonen et al., 2013; Peebo et al., 2015), the proteome might change throughout the cultivation and show decreased sugar uptake rates (Borirak et al., 2015; Peebo et al., 2015). Intracellular stress might therefore lead to decreased enzyme activity, such as a reduced activity in the phosphotransferase system, which is primarily responsible for sugar uptake (Deutscher et al., 2006). Neubauer et al. established a model where cellular capacities are limited as an effect of heterologous gene expression (Neubauer et al., 2003). As intracellular proteins are measured also within dry cell weight quantification it is important to separate the cell into a “functional cell part” (showing metabolic activity) and a “recombinant part” (being unable to show metabolic activity). Decreased enzyme availability might lead to high sugar accumulation in the fermentation broth, especially when exponential feeding is applied (Slouka et al., 2018a), as already stressed cells might additionally suffer from osmotic stress, triggering cell death (Slouka et al., 2019). It is shown that high sugar uptake rates tend to shift toward acetate formation, finally leading to decreased levels of production (Fragoso-Jiménez et al., 2019). Therefore, appropriate feeding rates beneath μ_max_ should be set to avoid overflow metabolism; however, acetate production can also be significantly reduced using engineered strains, as shown by Lara et al. (2008), Valgepea et al. (2010), Peebo et al. (2015), and Anane et al. (2017). Higher overall titers within *Pichia pastoris* are achieved when switching from static q_s_-based controls to a dynamic feeding strategy (Spadiut et al., 2014c). Feeding strategies using a static q_s_ control within *E. coli* might therefore need to be adapted.

#### Metabolic Burden and Its Correlation With IPTG

Declines in growth rates can be linearly correlated to the amount of recombinant protein produced (Scott et al., 2010). However, studies using IPTG as inducer have to take into account that this μ-decrease might result from the inducer itself, as IPTG is known to exhibit toxicity in host cells at long cultivation times (Dvorak et al., 2015). Malakar et al. showed that the growth rate declines in relation to higher amounts of IPTG used, which leads to higher recombinant protein translation rates (Malakar and Venkatesh, 2012). Transcriptomic results derived from fed-batch metabolism hint that essential genes needed for RPP are sequentially turned down when host cells are induced with IPTG, indicating that the metabolism switches toward a stationary phase (Haddadin and Harcum, 2005). As IPTG seems to be causing a high metabolic burden, it might not be a feasible inducer for long-term production.

#### How to Determine the Metabolic Burden

**Figure 1:**
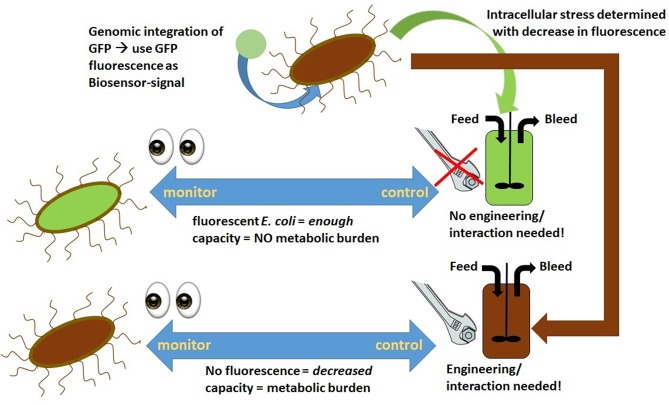
Stress determined via fluorescence measurement application as an on-line tool during chemostat cultivation to determine metabolic burden and, if necessary, intervene.

Operating at high specific feeding rates, such as q_s_ of 0.4–0.5 g/g/h, during the induction phase shows a trend of decreasing uptake “capacity,” which results in high extracellular sugar accumulation and increased cell death (Kopp et al., 2018; Slouka et al., 2019). Ceroni et al. aim to determine the metabolic load using a GFP (green fluorescent protein) cassette as an integrated marker protein to determine the “capacity” of cells (Ceroni et al., 2015). The capacity measurements of the cells match the theory established by Scott et al. even though the overlapping emission of GFP and m-cherry, used within this study, has to be taken into account more thoroughly (Scott et al., 2010; Ceroni et al., 2015). As metabolic burden is known to cause limitations in RPP (Heyland et al., 2011), the burden control system, also established by Ceroni et al. in a follow-up study, could soon be implemented in industry. The results thus indicate that *in-vivo* controlled cells exhibited higher capacity and showed better process performance than common, unregulated cells (Ceroni et al., 2018). As heat-shock promoters were significantly upregulated during recombinant protein expression, RPP is controlled *in vivo* using these promoters for capacity measurement. A dcas9 feedback control is used to adapt the protein expression due to the measured capacity. Application of burden–control systems in continuous cultivations, as demonstrated in Figure 1, would be an interesting approach to maintaining cells in a “stable capacity.” Furthermore, as GFP is used as a capacity marker within this system, at-line, and even online process controls using plate readers or flow cytometry could be established for this regulated strain.

### Intracellular vs. Extracellular Expression for *E. coli*

High metabolic burden is also often associated with the accumulation of intracellular proteins as the chaperones become overloaded and inclusion body formation can be observed (Fahnert et al., 2004; Ramón et al., 2014). As misfolded intracellular proteins are also known to suffer from long purification times and occasionally low refolding yields (Jungbauer and Kaar, 2007; Singh et al., 2015; Slouka et al., 2018b), the production of soluble extracellular proteins might be a feasible approach. Protein secretion, commonly performed in eukaryotic cells, might also be a key solution to reducing the metabolic burden; however, recombinant protein secretion is not easy to establish in bacteria (Berlec and Strukelj, 2013; Rosano and Ceccarelli, 2014). Still, RPP in the periplasm of *E. coli* has shown to yield promising results, especially when producing antibody fragments, making use of the oxidizing environment (Spadiut et al., 2014a; Kasli et al., 2019). To enable protein secretion in *E. coli*, engineering of the Sec-Pathway is commonly performed, leading to unfolded protein secretion (Mergulhão et al., 2005; Burdette et al., 2018). Alternatively, the SRP pathway can be used to secrete unfolded hydrophobic amino acid sequences while the twin-arginine pathway can be used to secrete folded proteins to the outer membrane (Berlec and Strukelj, 2013). As the amount of recombinant protein can be as high as 50% of the complete protein content of the cell, the RPP therefore sometimes might be limited by the secretion system itself (Rosano and Ceccarelli, 2014). Even though it is challenging to achieve extracellular RPP in *E. coli*, the company WACKER engineered a secretion system, called ESETEC, which has been upgraded to ESETEC 2 and is able to achieve high extracellular titers (Richter and Koebsch, 2017). Morra et al. investigated the phenomena of “non-classical protein secretion,” where cytoplasmic proteins can be secreted into the supernatant via mechanosensitive channels, triggering secretion via stress (Morra et al., 2018). Other studies, using triton, sucrose or glycine to secrete product into the supernatant, indicate a promising process strategy for fed-batch cultivations, releasing product into the fermentation broth before harvest and thereby reducing the purification time (Bao et al., 2016; Na et al., 2019). To implement a long-term process, constant, stress-free protein secretion is desired. Previous tests of recombinant protein secretion in chemostat experiments (operated at D = 0.1 h^−1^) show the feasibility of the study; however, the extracellular protein concentration dropped significantly over the period of cultivation (Selvamani, 2014). Although soluble protein production would be promising, it was shown that achieved titers were so far still within non-feasible ranges (mg/L to low g/L range), independent of the cultivation system (Mergulhão et al., 2005; Kleiner-Grote et al., 2018). Still we would like to hypothesize, with ongoing host engineering, that extracellular protein production within *E. coli* might be an interesting approach for the future.

### Enablers for Continuous Processing With *E. coli*

#### Process Controls in Fed Batch With Tech Transfer Possibility to Continuous Processing

Even though intracellular shifts might be noticeable (due to high metabolic burden put on the cells by RPP) the CQAs of the recombinant product have to be maintained constantly throughout the process (Rathore, 2009). We defined CQAs for an *E. coli* inclusion body fed-batch process, investigating IB (inclusion body) size, purity, and titer (Slouka et al., 2018a). Lower temperatures throughout the induction phase seemed to be favorable for RPP throughout this and other studies (Wurm et al., 2017a; Slouka et al., 2018a). This might be due to the high amount of energy needed to produce recombinant proteins. Host cells might shift intracellular fluxes toward toxic pathways in order to regenerate reducing equivalents and further cope with the energy demand needed. A reduction of cell densities, temperature, or separation of biomass growth and RPP might facilitate stability in continuous processes (Rugbjerg and Sommer, 2019). Enabling higher sugar uptake with engineered strains shows that specific productivity and specific sugar uptake rates were correlated in a bell-shaped curve; extreme sugar uptake rates lead to fermentative growth (Basan et al., 2015; Peebo et al., 2015; Basan, 2018; Fragoso-Jiménez et al., 2019). The metabolic load therefore has to be considered, and feeding strategies and model-based approaches that consider the shifts in the growth rate have to be established (Kopp et al., 2018). Schaepe et al. implemented a feeding strategy where the fed carbon is adapted to the control of the online monitored oxygen consumption rate (OUR) in order to avoid overfeeding (Schaepe et al., 2014). As cells exhibiting a high burden tend to shift toward acetate formation (Martínez-Gómez et al., 2012; Schaepe et al., 2014), it might be interesting to control onto pre-established online capacity measurements throughout long-term cultivations (Ceroni et al., 2015).

#### Promotor Systems Applied in Fed-Batch and Its Tech Transfer Potential Within Continuous Processing

The most frequently used system for RPP used in *E. coli* is still the Bl21(DE3) strain in combination with pET plasmids, which make use of the strong T7 promotor under the control of the lac promotor and its repressor (Studier et al., 1990; Rosano et al., 2019). Even though BL21 (DE3) is considered the state-of-the-art strain for RPP in *E. coli*, homologous recombination rates might occur, especially throughout long-running fermentations (Rugbjerg and Sommer, 2019). Therefore, other strains that experience a *recA* deletion, such as HMS-174, might be a good alternative to promote long-term stability, especially as high product titers have been considered to use this strain (Hausjell et al., 2018). Using the T7-induction system, induction can be performed with a simple inducer pulse as IPTG binds to the lactose repressor (Malakar and Venkatesh, 2012; Marbach and Bettenbrock, 2012). For standard fed-batch approaches this system works fine as high titers can be achieved within short time (Slouka et al., 2019); however, full induction of the T7-polymerase using IPTG puts a high metabolic burden on the host cells (Dvorak et al., 2015). The ptac promotor in combination with IPTG and lactose as an inducer allows the interesting approach of tunable protein expression, resulting in a lower metabolic burden put onto host cells (Marschall et al., 2016). A rather new approach, also making use of the T7-polymerase, is a double induction system, which shuts down the *E. coli* RNA-polymerase, inhibiting host mRNA production (Lemmerer et al., 2019). Therefore, the total energy flux derived from fed carbon could be used for RPP. Regulating this system in a tunable way, with a switch-on/switch-off strategy might be an interesting approach for chemostat experiments. A further method to separate rapid growth from RPP might be the utilization of stress-regulated promoters. Making use of phosphate limitation (phoA-promoter), cAMP/CRP-system regulations (cap promoter with MglD-Repressor), and other promotor systems could enhance a more resistant subpopulation in chemostat cultivations, producing at a low but steady level (Neubauer and Winter, 2001). Further positive regulation systems, also being referred to as tunable, are the araBAD and the rhamBAD induction systems, which are induced by arabinose and rhamnose, respectively (Khlebnikov et al., 2002; Wegerer et al., 2008). Tunable protein expression systems, discussed in detail by Marschall et al. (2016), allow for the control of expression rates and should therefore ease control strategies for continuous processes. However, the major advantage of *E. coli* is still its relatively cheap production, and the utilization of highly expensive inducers in a continuous production scale might not be feasible as media costs would rise drastically. When comparing costs of “tunable” inducers in *E. coli* with the methanol inducible AOX system of *P. pastoris*, it is obvious that a less expensive induction system needs to be found (Mattanovich et al., 2012; Spadiut et al., 2014b). Lactose, also referred to as being be a tunable inducer (Neubauer et al., 1992; Neubauer and Hofmann, 1994), has been shown to boost soluble intracellular protein concentrations in *E. coli* fed-batches so far (Wurm et al., 2016, 2017c). For white biotechnology approaches, lactose could be purchased cheaply as side products of the milk and cheese industry (Viitanen et al., 2003). The implementation of lactose as an inducer in continuous cultivations could therefore lead to interesting and affordable approaches.

#### Engineering on a Genomic Level and Its Tech Transfer Potential Within Continuous Processing

Plasmid technology is still state of the art for RPP in *E. coli* (Rosano et al., 2019). Selection is commonly employed using antibiotics, though a drug substance (= DS) produced in *E. coli* needs to be free from antibiotics (Silva et al., 2012). As high copy number plasmids, frequently used in industry, put a high metabolic burden onto the host, the regime of plasmid-based systems might slowly fade out (Fink et al., 2019). The production load here is usually used to trigger an escape rate, leading to diverse subpopulations within the cultivation system (Rugbjerg et al., 2018). Non-productive subpopulations might arise from diverse point mutations or reallocations, emerging at an escape rate as high as 10^−5^-10^−8^ per generation in plasmid-based *E. coli* systems (Rugbjerg and Sommer, 2019). As high burden might also lead to high amounts of recombineering, the genomic integration of the gene of interest (GOI) would reduce host stress as copy numbers can easily be controlled, and no antibiotic selection is required (Mairhofer et al., 2013). Introducing the GOI into the host can be performed by CRISPR-Cas9 strategies or via a recombineering method (Reisch and Prather, 2015). While CRISPR-Cas9 selection functions via a double-strand cleavage, recombineering needs antibiotics resistance to select the GOI. This antibiotic selection, however, can be cured in an ongoing step (Biswas et al., 2012; Chung et al., 2017; Reisch and Prather, 2017). Striedner et al. performed chemostat cultivations with *E. coli* cells, producing GFP, where the gene was integrated into the genome (using the recombineering method). However, production was not stable for 10 generation times (Striedner et al., 2010). Within a recent study it was shown that the location is highly dependent on the expression of the GOI (Englaender et al., 2017), which probably explains the difficulties in stability. In a different study, the integration of large DNA-fragments, like complete pathways, was performed. This is sometimes difficult to reproduce within different strains (Chung et al., 2017; Englaender et al., 2017). On the other hand, Wang et al. showed that genomic engineering of the complete mevalonate pathway exceeded plasmid-based mevalonate production (Wang et al., 2016).

An interesting approach was performed by Rovner et al. who engineered a codon-optimized *E. coli*, creating auxothrophy for phenylalanine-derived amino acids (Rovner et al., 2015). Coupling the expression of essential genes to synthetic amino acids, no escape rate could be monitored within 20 days of cultivation. Even though the selection criteria of synthetic amino acids might be much more effective than common selection systems, the amount of synthetic amino acids needed for feeding at an industrial scale might not be feasible (Stokstad, 2015). This results in the need for a selection mechanism being “cheap enough” to boost selection criteria also at large volumes. Strategies such as connecting cell growth with product formation would increase the fitness of the subpopulation needed for RPP and further enhance overall yields (Buerger et al., 2019). Furthermore, the idea of a two-way selection was shown to improve the host, as exemplarily shown for an *E. coli* naringenin producer, which was increased by a factor of 36 (Raman et al., 2014). As negative selection does delete escaped subpopulations, positive selection can help to find high-productive mutations. The toggling selection mechanism, which regulates between positive and negative selection, could be the way to go to create strains needed for long-term stable production systems.

Trying to reduce the host's metabolic burden, the usage of protein secretion or the integration of the target sequence into the genome seem to be promising tools. Further host engineering might enable the continuous production of recombinant proteins in *E. coli* by eliminating the needs of antibiotics. However, it seems like neither industry nor academia is there yet, as a complete understanding of the interactions between the GOI and integration site is still missing and needs to be tested (Englaender et al., 2017). Strains commonly employed in continuous cultivations are screened for maximum production within short cultivation times (Jia and Jeon, 2016). Even though it is rather time consuming, it might be necessary to screen for long-term stable strains in order to find appropriate candidates for the implementation of continuous cultivations.

## Achievements in Continuous Upstream Applications With *E. coli*

Basing their description of bacterial growth on that which was established by Monod (Monod, 1949b), the term “chemostat” was defined by Novick and Szilard in the 1950s (Novick and Szilard, 1950a). Many physiological characterization studies have been carried out in *E. coli* chemostat processing since then, and the behavior of cells is understood in more depth nowadays (Wick et al., 2001; Peebo et al., 2015; Kurata and Sugimoto, 2018). As RPP in continuous cultivation systems in *E. coli* leads to uncharacteristic intracellular fluxes (Peebo and Neubauer, 2018), there is a high demand in process analytical tools to monitor and control cultivation systems (Esmonde-White et al., 2017; Vargas et al., 2018; Zobel-Roos et al., 2019). Within this section we therefore discuss the physiological characterizations of *E. coli* chemostat cultures; process analytical tools (PAT) implemented in *E. coli* processes and their applicability within continuous systems; and engineering screws to optimize long-term *E. coli* cultivations.

### Physiological Characterization of *E. coli* Chemostat Cultures

The introduction of steady-state cultivation systems back in that 1950s was applied to ease research approaches, such as estimating mutation rates (Monod, 1949a; Novick and Szilard, 1950a,b, 1951). The “competitive ability” of *E. coli* was investigated already back in the 1980s, using a molecular clock principle, and showed that most changes in population fitness occur within the first 200 h of a cultivation (Dykhuizen and Hartl, 1981). Proteome changes in *E. coli* chemostat cultivations were monitored when switching from glucose-limiting conditions to glucose excess after 500 h of cultivation (Wick et al., 2001). This study shows that the proteome can be distinguished in a short and in a long-term response (Wick et al., 2001), indicating that changes in the transcriptome could be monitored in long-term cultivations even without the production of recombinant proteins (Peebo and Neubauer, 2018). Chemostat cultivations, performed with different dilution rates, revealed that RPP is fairly constant within an induction time of 6 h (Vaiphei et al., 2009). Q_p_ can be correlated to the growth rate, which is in accordance with other studies and independent from the cultivation mode (Peebo and Neubauer, 2018; Slouka et al., 2019). The phenomenon of growth-dependent production shows a need for investigating intracellular pathways in more detail (Valgepea et al., 2013). A kinetic model, developed by Kurata et al. describes intracellular carbon pathways down to the TCA-cycle and can be verified for non-induced cultivations (Kurata and Sugimoto, 2018). Still, it has to be taken into account that intracellular fluxes tend to shift, once RPP is induced, as the maximum growth rate decreases due to limited host cell capacity (Scott et al., 2010; Heyland et al., 2011; Ceroni et al., 2015). As seven volume changes in a steady-state recombinant protein production in single-vessel *E. coli* cultivations are regarded as a steady-state (Vemuri et al., 2006), studies in this field are tricky to implement, as cultivations might suffer from unexpected metabolic shifts before reaching a “steady-state” mode. Characterizing intracellular fluxes via a metabolome analysis indicates a high diversity occurring between different strains (Basan, 2018; McCloskey et al., 2018). The metabolome analysis state, which we use to deal with a highly complex system as different intracellular pathways are up- or downregulated, is very dependent on the host and the target protein (McCloskey et al., 2018). As shifts already occur without RPP, showing that cells try to adapt to the environment in chemostat cultivation, we hypothesize that the production of recombinant proteins will increase these shifts to a maximum.

### Process Analytical Tools (PAT) in *E. coli* Cultivations

Defining a certain time span of a continuous production as a lot number has opened up the possibility of releasing products of long-term processes, providing constant CQAs of each lot number independently from process time (Allison et al., 2015). In process realization, analytical methods often create a bottleneck as they are highly time consuming [taking sample preparation and data treatment into account (Pais et al., 2014; Gomes et al., 2015; Sommeregger et al., 2017)]. At-line analytics might provide useful information (Lee et al., 2015); however, process control in continuous manufacturing should be implemented using online signals (Rathore et al., 2010), if possible, making use of PAT (process analytic tools) (Rathore, 2015; Vargas et al., 2018). Process controls using a “digital twin” are the way to move toward Biopharma 4.0 (Nargund et al., 2019), but they still need to be established for long-term *E. coli* cultivations. Within this monitoring strategy, first brought to public by NASA (Rosen et al., 2015), real processes are represented by virtual simulations and are continuously fed with all process parameters and sensor results monitored throughout the process (Zobel-Roos et al., 2019). Using model-based controls in combination with online data transmission, process variances can be predicted, and operators can intervene to keep processes going (Zahel et al., 2017a; Steinwandter et al., 2019).

Some online process controls implementing PAT are nowadays carried out with Raman spectroscopy measurements in cell culture cultivations (Abu-Absi et al., 2011; Lewis et al., 2018; Nagy et al., 2018). Esmonde-White et al. found that Raman spectroscopy is already applied in GMP facilities (good manufacturing practice) for monoclonal antibody (mAB) production (Esmonde-White et al., 2017). Raman spectroscopy can be found in diverse fields, ranging from bacterial impurity measurement of water (Li et al., 2017) to surface-enhanced Raman spectroscopy (SERS), which can be used to determine bacterial contaminations in cell cultures (Esmonde-White et al., 2017). Teng et al. monitored stress reactions using Raman spectroscopy on a single-cell bacterial level (Teng et al., 2016), and overflow metabolites in an *E. coli* fermentation were monitored by Lee et al. using online Raman spectroscopy (Lee et al., 2004). Nevertheless, process controls for RPP in *E. coli* have not been established until now.

As biomass provides important information about *E. coli* process characteristics (Slouka et al., 2016), online dry cell weight estimations have been performed with online microscopy and back-scattered light (Marquard et al., 2017; Mühlmann et al., 2018). Since scattered light analysis is difficult to implement on a large scale, and microscopy might suffer from the use of complex media, process control implementation is tricky. At-line flow cytometry measuring viable cell concentration can provide useful information to determine states throughout a cultivation (Langemann et al., 2016). Using GFP reporter strains, it has been shown that the adaption of glucose pulses in chemostat cultivation could be monitored using flow cytometry analysis (Heins et al., 2019). Baert et al. also used flow cytometry as a tool to determine variations in phenotypes (Baert et al., 2015). Cell filamentation and its correlation with high productive subpopulations are also monitored with flow cytometry in fed-batch cultivations (Fragoso-Jiménez et al., 2019). Measuring filamented populations via PI-staining showed that the filamented subpopulation had enhanced PI uptake, forming the so-called red but not dead phenotype (Shi et al., 2007; Davey and Hexley, 2011). Sassi et al. presented an interesting follow-up approach, which showed the possibility of monitoring and controlling subpopulations with online PI staining being detected with online flow cytometry in chemostat cultivations (Sassi et al., 2019). The red but not dead phenotype population can be regulated at a constant level of 10% using a cultivation system called the “segregostat,” which applies starvation or glucose pulses. Still, the feasibility of this cultivation mode needs to be tested for continuous recombinant protein formation, and proper online dilution systems need to be established in order to dilute high cell density cultivations (Langemann et al., 2016). Nevertheless, the applicability of an online flow cytometer as a PAT for continuous microbial fermentations is clearly needed in order to characterize heterogeneous differences throughout cultivation (Delvigne et al., 2017). The determination of viable biomass with rheological measurements (Newton et al., 2016) could also be implemented in an at-line application. Soft-sensor cultivation for recombinant GFP production was also established using NIR-signals and at-line HPLC to control metabolite accumulation by Gustavsson and Mandenius (2013). Applications like this could be of particularly high interest for continuous cultivations. High throughput metabolite quantification such as the RP-LC-MS/MS method by McCloskey et al. may help to get an at-line “host response” (McCloskey et al., 2018). Understanding intracellular dynamics on a single-cell level might additionally help to characterize different phenotypes occurring throughout the cultivation (Leygeber et al., 2019). Nevertheless, further understanding on a transcriptome and proteome level will be needed to shed more light on intracellular fluxes. Therefore, characterizing the intracellular fluxes throughout the production of recombinant molecules, with metabolites being measured at-line, is the way to develop adequate process control strategies. Table 1 gives a short overview of monitoring tools developed for *E. coli* cultivations up until now, and these can be implemented in an online control mode for continuous cultivations.

**Table 1:** PAT tools established in *Escherichia coli* or with promising prospects for establishment.

**Monitoring tool**	**Scale & cultivation mode**	**Application**	**Host & target product**	**References**
Raman-spectroscopy	Fermentation 2.5 L, batch mode	Measuring of glucose, lactate, formate, acetate	*E coli* ATCC31883, for phenylalanine production	Lee et al., 2004
NIR (near infra-red) spectroscopy	Fermentation 2 L, batch mode	Glucose, glycerol, ammonium, and acetate measurement with at line NIR	*E. coli* ML308 & *E. coli* W310	Schenk et al., 2008
*In situ* microscopy (ISM)	Fermentation 2 L, fed-batch	Cell concentration measurement up to 70 g/L	*E. coli* BL21(DE3)	Marquard et al., 2017
Electrochemical impedance spectroscopy (EIS)	Fermentation, 10 L, fed-batch	increase in the double layer capacitance at low frequency can be correlated to biomass growth; offline and online analysis	*E. coli BL21 DE3, horseradish peroxidase production*	Slouka et al., 2016
Viscosity monitoring for cell lysis	Fermentation 5 L, fed batch	DNA release due to cell lysis increases viscosity up to 25%; cell lysis can be determined with at-line rheological measurement	*E. coli* W3110, for Fab production	Newton et al., 2016
Scattered light for predication of burden	Biolector plate & RAMOS shake-flask	Scattered light is calculated to oxygen transfer rate & biomass prediction on burden	*E. coli* BL21 (DE3) for cellulose and fluorescent protein FbFP production	Mühlmann et al., 2018
Fermentation off-gas analysis to determine metabolic state	Fermentation 15 L, fed batch	Online Oxygen transfer-rate monitoring, Sugar accumulation/Overfeeding can be correlated with decreased signal of OTR	*E. coli BL21 DE3, sfGFP production*	Schaepe et al., 2014
At-line flow cytometry for bacterial cell lysis	Fermentation 20 L, fed batch	Combination of 2 fluorescent stains [DiBac_4_(3)) & Rh414] showed that viable cell counts from FCM measurements could be correlated with viable CFUs	*E. coli* NM522, no RPP	Langemann et al., 2016
Online flow cytometer control—“segregostat”	Fermentation 2 L, chemostat	Monitoring & control of PI-positive phenotype with online FCM and control via glucose pulses or starvation	*E. coli* JW2203-1 Δ*ompC*, no RPP	Sassi et al., 2019
Soft-sensor-sensor PAT application (NIR + at-line HPLC)	Fermentation 10 L, fed batch	NIR *in situ* probe & at-line HPLC for measuring overflow metabolites to control metabolites at a set level	*E. coli* HMS174, GFP production	Gustavsson and Mandenius, 2013
Full-rip (RP-LC-Ms/Ms) for metabolome characterization	Shake flask cultivation, 25 m L	Offline measurements of metabolomics with RP-LC-MS/MS; determining 100 metabolites within 5 min; differences in the central, amino acid, nucleotide, and energy pathways were found	*E. coli C*, *E. coli Crooks*, *E. coli DH5a*, *E. coli W*, *E. coli W3110*, *E. coli BL21 (DE3)*, *E. coli K-12-MG1655*	McCloskey et al., 2018
Online scattered light measurement for IB measuring at 625 nm	Shakeflask cultivation	180° measurement for morphological, opacity & color change	*E. coli*, BL21 (DE3) for GFP & hLIF production	Ude et al., 2016

Mammalian cell cultivations for RPP already make use of RAMAN- and NIR-signals (Iversen et al., 2014; Berry et al., 2015; Esmonde-White et al., 2017; Li et al., 2017), and even have implemented signal-derived feedback controls (Li et al., 2013; Craven et al., 2014). As the major advantage of microbial processes compared to mammalian cell line cultivations is the cheap manufacturing, the devices and detectors needed for Raman and NIR spectroscopy are possibly too high in price to establish microbial process controls until now (Rathore, 2014). With cheaper analytical devices being developed, the future might show a new trend in process controls within *E. coli*.

### Engineering Screws to Optimize Long-Term *E. coli* Cultivations

As different continuous cultivation modes for microbial systems have been discussed by Adamberg et al. (2015), we will focus on continuous cultivation systems, which are feasible for RPP. Fermentations with *E. coli* need high gas flows and high stirrer velocities to accomplish the respiratory demand of the host cells (Schaepe et al., 2014). Scale-up via k_LA_ is well-understood and can be performed without observing a large-scale yield decrease (Junker, 2004; Islam et al., 2008). The development of continuous cultivation systems using mini-reactor systems is therefore a feasible approach (Lis et al., 2019), especially as continuous culture development is highly time and media consuming (Schmideder et al., 2016). Multiple small-scale reactors can be operated in parallel, increasing the number of experiments (Schmideder et al., 2015). Furthermore, it opens up the possibility to screen multiple candidates in a high throughput manner (Bergenholm et al., 2019), which might be necessary to develop suitable strains for continuous manufacturing.

**Figure 2:**
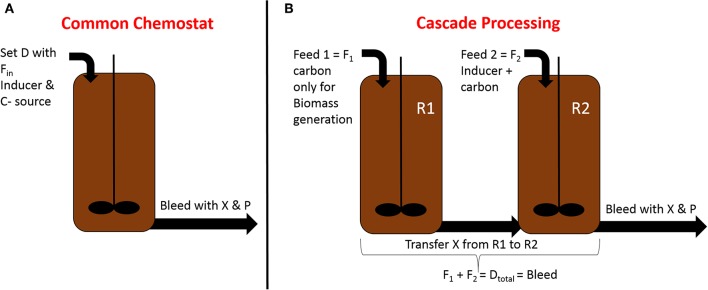
Comparison of common chemostat processing in **(A)** with the established cascaded processing system in **(B)**; different process parameters for biomass growth and RPP can be adjusted in each reactor using the cascaded system, optimizing RPP; X, Biomass; P, Product; D, dilution rate; R1, reactor 1 for biomass production; R2, reactor 2 for recombinant protein production.

To overcome stability issues in long-term RPP within *E. coli*, Schmideder et al. implement a cascaded system, pictured in Figure 2, where two stirred tank reactors were operated in parallel at different conditions (Schmideder and Weuster-Botz, 2017). Steady-state production within an *E. coli* plasmid-based system could be reached for two residence times within this study. Higher dilution rates seem to be beneficial for RPP in cascade cultivations, which is in accordance with chemostat- and fed-batch experiments (Vaiphei et al., 2009; Peebo and Neubauer, 2018; Slouka et al., 2019). As product formation within the host cells is a time-dependent process in *E. coli*, there is a trade-off between maximum productivity and steady state production. Maximum q_p_ in fed-batch usually is usually achieved after ~10 h of induction when maintaining a growth rate of μ = 0.1 h^−1^ (Slouka et al., 2018a). As occurring cell lysis and decreased host capacity towards the end of a fed-batch cultivation possibly lead to “non-productive” subpopulations, continuous cascaded systems operating at higher dilution rates, washing out cells before achieving maximum productivity seems to be beneficial to achieve stable productivity in two-compartment systems.

An interesting approach on the pharmaceutical horizon might be moving away from only RPP and move onto recombinant mRNA production (Pardi et al., 2018; Zhang et al., 2019). As *E. coli* is a well-characterized host, with cheap cultivation possibilities (Gupta and Shukla, 2017; Wurm et al., 2017c; Slouka et al., 2018b; Rosano et al., 2019), recombinant mRNA production might soon be performed within *E. coli*. Establishing continuous cultivation systems for recombinant mRNA is yet to come; however, we would suggest that process technology should be ready for such implementations.

## The Continuous Downstream Processing of Intracellular Proteins

The continuous downstream processing of extracellular proteins derived from cell culture cultivations (mostly monoclonal antibody production) is already implemented in some branches of the pharmaceutical industry (Vogel et al., 2012; Ötes et al., 2018). Proteins can be separated from impurities at high efficiency either with SMB (simulated moving bed)-chromatography or continuous membrane chromatography (Shekhawat and Rathore, 2019). Workflows and the processing of proteins in a continuous mode have recently been discussed by Jungbauer in more depth (Jungbauer, 2013). While extracellular proteins are separated via centrifugation from the host cells, are captured as they go, and then purified and polished, intracellular proteins need more downstream unit operations to achieve the same quality attributes. As stable product formation within *E. coli* in a continuous mode has so far been difficult to realize; the difficulties of continuous purification of intracellular protein have not been discussed in much depth (Peebo and Neubauer, 2018). Therefore, we will give an update on the newest achievements in continuous downstream processing for intracellular proteins derived from *E. coli*.

Purification of intracellular proteins generally starts with a centrifugation step, separating biomass from the supernatant (Jungbauer, 2013). Simulations of a disc stack centrifugation applied in a continuous mode have been done and technical realization is feasible for continuous application (Chatel et al., 2014). Ongoing cell disruption is performed with high-pressure homogenization, as high-pressure homogenization is the only scalable form of cell disruption (Balasundaram et al., 2009; Lin and Cai, 2009). Homogenizers can be operated at high velocities; realizing cell disruption within one passage and the implementation of a continuous cell disruption mode is rather easy (Barazzone et al., 2011). The following centrifugation step, separating cell debris from soluble proteins, is also feasible in a continuous mode with techniques available nowadays (Palmer and Wingfield, 2012; Chatel et al., 2014). Even though IBs suffer occasionally from low yields throughout refolding unit operation, product purity was found to be as high as 80% after differential centrifugation, which would lead to a decreased demand in chromatography (Jungbauer, 2013).

It was shown that IB processing can be boosted when operating the notorious refolding step in a continuous mode (Wellhoefer et al., 2014; Walch and Jungbauer, 2017). Within different studies, the continuously refolded lysozyme exhibited higher refolding yields than commonly performed batch/fed-batch refolding (Farshbaf et al., 2002). As a lysozyme is known to have high refolding yields (Sakamoto et al., 2004; Rathore, 2013) when compared to many other proteins, yield enhancement by continuous refolding still needs to be verified with different proteins (Yamaguchi and Miyazaki, 2014; Pieracci et al., 2018). Still, Schlegl et al. simulated the refolding efficiency in continuous stirred-tank reactors, and the results showed that higher refolding yields could be achieved in continuous applications when compared to batch processing (Schlegl et al., 2005). As dilutions in batch refolding unit operation increase the refolding yield (Vemula et al., 2015), huge volume capacities are needed (Yamaguchi and Miyazaki, 2014). Wellhoefer et al. further mark that continuous refolding could save up to 98% of the refolding buffer applied within a process (Wellhoefer et al., 2014). Mild solubilization techniques were discussed in more detail by Singh et al. and are also known to enhance product yield depending on the target protein (Singh et al., 2015). Applying continuous solubilization in a plug-flow reactor system and passing the solubilized protein on to a continuous refolding unit operation seems to be the holy grail in continuous IB processing, as reactor volumes can be reduced and efficiency can be enhanced.

Endotoxin removal represents a further issue in bacterial processing as purification is dependent on the target protein (Hyun et al., 2009; Lopes et al., 2010). Depending on the target protein size, lipopolysaccharide purification can be performed with an ultrafiltration step (Pieracci et al., 2018). However, establishing a clean cutoff might be tricky as endotoxins usually are in the size-range of 10–20 kDa (Hyun et al., 2009). Two phase applications for endotoxin removals might also be challenging to operate in a continuous mode as long residence times are needed to extract endotoxins (Lopes et al., 2010). This leaves chromatography as the most efficient operation mode to continuously purify proteins from lipopolysaccharides (Lin et al., 2005). A case study is shown by Kateja et al. where IBs are purified in a continuous mode using two different chromatography systems, each of them using three stacked columns, operated in a counter current system (Kateja et al., 2017). Defined CQAs can be kept at a constant level within a processing timeframe of 26 h, but analysis is performed after the process as the PAT for each unit operation still needs to be established. Progress in downstream PAT development is carried out by using a model for real-time monitoring, and this was established using standard measurements such as, pH, conductivity, and UV-VIS absorption as a model input (Sauer et al., 2019). Online prediction of host cell proteins, DNA impurities, and an estimation of the protein content throughout the purification step is possible using this model. Methods of establishing more model-based PATs and using process parameters as inputs are yet to be established for each unit operation. By understanding the effects of process parameters onto product quality, a digital process can be simulated, and process control can be implemented, making use of digital twins, also in downstream processing (Zobel-Roos et al., 2019).

## Discussion

Up until now, continuous RPP in a one-compartment system (Adamberg et al., 2015); using *E. coli* as a host is still lacking in stability issues (Peebo and Neubauer, 2018). It was always thought that *E. coli* fed-batch processes were already fully characterized, but occurring stress responses by host cells might have never been monitored until now due to the short time span of fed-batch cultivations. Trying to establish a time-independent cultivation system, we have to go back to a black-box model as, so far, unexplainable effects have occurred throughout long-term cultivations. Even though *E. coli* exhibits slightly slower mutation rates than mammalian cells (10-^10^/base pair per generation for *E. coli*; 10-^8^/base pair per generation for mammalian cells), the high growth rate of bacteria and therefore the high amount of generation times might explain the potential shifts in continuous systems with *E. coli* (Rugbjerg and Sommer, 2019). We hypothesize that the same shifts in transcriptomes and proteomes would occur through continuous processes with mammalian cells if processes ran long enough to achieve a comparable number of generation times. However, we believe that, once intracellular fluxes can be monitored and are understood in greater depth, we can adapt control strategies to keep subpopulations at a constant level, which has been exhibited for uninduced systems already (Sassi et al., 2019). Even though single-stage cultivation systems might not be ready yet for continuous RPP in *E. coli*, the previously mentioned two-compartment system showed stable RPP with *E. coli* (Schmideder and Weuster-Botz, 2017). The feasibility of a continuous inclusion body downstream process has also been demonstrated (Kateja et al., 2017); therefore, it seems like process technology is ready for implementing RPP in a continuous mode, using *E. coli* as a host. Still, we believe that more work needs to be done in the physiological characterization and implementation of process analytical tools to establish a more robust process control. Current problems in the continuous processing with *E. coli* and solutions are thus summarized and highlighted in Table 2. Once online monitoring devices are ready, we only need a change of mind-set and a switch from batch processing toward a fully integrated continuous process, and there are indeed multiple benefits to performing continuous RPP within *E. coli*.

**Table 2:** Current problem states and approaches to move on toward continuous processing with *E. coli*.

**Chapter**	**Problem state**	**Effect**	**Current approach**	**Proposed next step**	**References**
The current state in *E. coli* fed-batch process understanding and its tech transfer potential to continuous processing	Metabolic burden & measurement of such	Intracellular stress, decreasing viable cell concentration & decreased levels of RPP	Time of induction is adapted to achieve high RPP; new *in vivo* burden control system tunable promotor systems, extracellular protein formation	Establish online burden control using online PAT applications → biological system = complex → get process understanding establish → robust process	Neubauer et al., 2003; Scott et al., 2010; Ceroni et al., 2018; Fragoso-Jiménez et al., 2019
	Extracellular protein production	Reduce complexity and time of protein purification	Chemostat cultivations with extracellular protein production suffers from q_p_ decrease over time	Establish better secretion systems, protein → strain engineering; q_p_ needs to be comparable to intracellular concentrations	Mergulhão et al., 2005; Selvamani, 2014; Kleiner-Grote et al., 2018
	Tunable promotor systems	Reduce metabolic burden on host cells during RPP	Arabinose & rhamnose can be used as tunable induction systems but are expensive in utilization	Lactose might be used as a tunable inducer; Development of new tunable inducer systems feasible for industrial approaches	Marschall et al., 2016; Wurm et al., 2017a,b,c
	Genomic integration	Stable product formation due to reduced burden	Integration of GOI with Recombineering/ CRISPR-Cas9; integration of complete pathways	Understand effects between GOI and integration site → need screening approaches for long-term stable production	Reisch and Prather, 2015; Chung et al., 2017; Englaender et al., 2017
The achievements in continuous Upstream applications with *E. coli*	Instable product formation in chemostat	Product formation decreases due to instable metabolome	Parallelization, Cascade processing;	Analysis and characterization of metabolome & transcriptome investigate & understand intracellular shifts; move on toward different feeding approaches; optimization of Cascade-processing;	Schmideder and Weuster-Botz, 2017; Peebo and Neubauer, 2018; Bergenholm et al., 2019; Sassi et al., 2019
	Lack of proper PAT in Upstream processing	Process control via at-line/offline measurements → time-delay in control → suffer from process variances	compare to Table 1 for PAT approaches	Monitoring of subpopulations establish → population controls with online flow cytometry; establish new process control with different spectroscopies like EIS;	Slouka et al., 2016; Esmonde-White et al., 2017; McCloskey et al., 2018
The continuous downstream processing of intracellular proteins	Lack of proper PAT in Downstream processing	Process control via at-line/offline measurements time-delay in control& quality check longer times to market	Offline sampling, model-based approaches	Monte-Carlo Simulations in order to find out criticality of each step Downstream = cell free, non-biological (ease in prediction) establish digital twin control	Wellhoefer et al., 2014; Kateja et al., 2017; Zahel et al., 2017b; Sauer et al., 2019

## Author Contributions

JK, CS, OS, and CH drafted the manuscript. JK and CS performed the literature research while OS and CH gave valuable scientific input.

### Conflict of Interest

The authors declare that the research was conducted in the absence of any commercial or financial relationships that could be construed as a potential conflict of interest.
